# Minimally invasive intrathoracic negative-pressure therapy and flexible thoracoscopy (FlexVATS) for patients with pleural empyema

**DOI:** 10.1038/s41598-023-37961-w

**Published:** 2023-07-05

**Authors:** Björn-Ole Stüben, Gabriel A. Plitzko, Julia Sauerbeck, Philipp Busch, Nathaniel Melling, Matthias Reeh, Jakob R. Izbicki, Thomas Rösch, Kai Bachmann, Michael Tachezy

**Affiliations:** 1 Department of General, Visceral and Thoracic Surgery, University Medical Center Hamburg-Eppendorf, Martinistraße 52, 20246 Hamburg, Germany; 2 Department of Diagnostic and Interventional Radiology and Nuclear Medicine, University Medical Center Hamburg-Eppendorf, Martinistraße 52, 20246 Hamburg, Germany; 3 Department of Interdisciplinary Endoscopy, University Medical Center Hamburg-Eppendorf, Martinistraße 52, 20246 Hamburg, Germany

**Keywords:** Health care, Infectious diseases, Respiratory tract diseases

## Abstract

To determine whether a new surgical method using a flexible endoscope (FlexVATS) to perform sparing debridement and apply negative-pressure therapy without extensive decortication may be an alternative treatment option for empyema. Surgical treatment of pleural empyema is associated with considerable postoperative complications and mortality rates, and alternative treatment options are being explored to improve patient outcomes. This was a prospective case series. Seventeen consecutive patients treated with FlexVATS between February 2021 and August 2022 were included in the study. Only patients for whom FlexVATS was the first therapeutic intervention for pleural empyema were included. Treatment success, defined as infection resolution, was the primary endpoint of the study. The secondary endpoints were length of hospital stay, 90-day mortality, and empyema cavity volume reduction. Patients who had previously been treated for pleural empyema by either drainage or surgery were excluded. The trial was performed as a single-centre study at a tertiary medical centre in Germany. In total, 17 patients with pleural empyema were included in the study. The median (IQR) duration of vacuum treatment was 15 days (8–35 days). Twelve of the 17 (71%) patients were successfully treated, and a significant reduction in the empyema cavity volume was observed. 41% of the dressing changes were performed outside the operating room. Compared with a historic cohort of conventionally treated patients (decortication via VATS or thoracotomy), the 90-day mortality rates tended to be lower without reaching statistical significance. Three patients (18%) died in hospital during treatment. No negative pressure-therapy-related complications were observed. FlexVATS therapy is a promising alternative therapy for both healthy and debilitated patients with pleural empyema. Larger randomised trials are required to validate this treatment option.

## Introduction

In the last 20 years, a continuous rise in cases and mortality rates have been reported for pleural empyema^1–4^. Older age and multimorbidity are risk factors for pleural empyema, with multimorbid patients twice as likely to develop pleural empyema^5^. There is a reluctance to treat early stage pleural empyema using surgical methods because of concerns regarding the patient’s ability to undergo surgery or tolerate single-lung ventilation impacting treatment outcomes.

The goal of surgical therapy is complete cleaning of the pleural cavity and re-expansion of the lungs. It is agreed upon that surgical therapy is necessary when adequate irrigation of the pleural cavity via thoracostomy drainage is complicated by fibrous septations, and recent therapy recommendations lean towards earlier surgical intervention^3,6^. The condition of the pleural space as well as the condition of the patient often dictate the surgical approach. Video-assisted thoracoscopic surgery (VATS) should be attempted whenever possible, given its proven benefits compared to open thoracotomy in terms of postoperative outcomes, such as improved postoperative pain control, shorter treatment times, and reduction in 30-day overall mortality^7,8^. A recent meta-analysis comparing thoracotomy to VATS decortication showed no significant differences in mortality rates or recurrences between the two treatment methods, with a shorter postoperative hospital stay for patients treated with VATS^9^.

Extensive decortication is an integral part of surgical therapy for advanced-stage empyema. However, decortication can come with a variety of complications which can prolong the treatment course and in multimorbid and elderly patients can even be life-threatening^10^. These complications include septic shock due to bacteraemia following decortication, intra- or post-operative haemorrhage, and persistent air leaks. In patients who are unfit to undergo decortication or tissue flap placement, open thoracic window (OTW) treatment may be necessary as a rescue procedure^7^.

Therefore, new treatment methods that minimize surgical trauma while providing source control in patients with pleural empyema are required.

Vacuum therapy as a treatment option for pleural empyema was first published in 2006^11^. Our group has recently published a systematic review and analysis of this treatment alternative^12^. While most studies described the technique as a form of volume reduction for patients who had undergone OTW for the treatment of empyema, some groups have reported the use of this treatment as the first option in primary cases of empyema^11,13–16^. This method appears to be a safe and successful alternative to standard surgical procedures. Based on this evidence, we further developed the technique by combining (A) minimally invasive techniques, using a flexible endoscope (FlexVATS) combined with rigid instruments, and (B) negative-pressure therapy with sponge material and open pore film application via small incisions.

The goal was to reduce surgery-related morbidity and septic complications by minimizing surgical trauma using small incisions and limiting treatment solely to the parts of the lung affected by empyema. Only local debridement and no decortication were performed, with the theory that the frequently seen bacterially triggered septic shock could be reduced or even prevented. Furthermore, we sought to reduce or avoid complications that often accompany extensive decortication, such as hemorrhage and persistent pulmonary air leaks as well as sepsis, with the latter being potentially lethal for older and debilitated patients^10^.

In contrast to conventional VATS with a rigid and straight fiberoptic, where access to the often small and winding parts of the empyema via the drainage channel is not possible without opening the complete pleural cavity, with flexible endoscopy a tool exists with which it is possible to reach all areas of the empyema cavity. This technique has already been validated for the management of anastomotic leakages after gastrointestinal surgery^17–19^.

We present the method and results for our series of patients treated with this method and carry out a critical analysis of the benefits, drawbacks, and potential usage in different stages of empyema and various degrees of comorbidities. In addition, we compared our data with those of patients treated using conventional VATS or open thoracotomy.

## Material and methods

### Data collection

All patients undergoing FlexVATS for pleural empyema at the Department of General, Visceral, and Thoracic Surgery at the University Medical Centre, Hamburg, Germany from February 2021 to August 2022 were included in a prospective database. All patients with a laboratory verified (via aspiration or drainage placement) and limited pleural empyema regardless of its origin were screened for the treatment modality. Preferably multi-morbid patients were included after an extensive explanation of the procedure and its potential advantages and disadvantages, and explicit consent was obtained from all subjects. All procedures were performed by or under the direct supervision of a senior surgeon (MT).

The medical records of all participating patients were retrospectively analysed. Preoperative demographic data, comorbidities, and causes of empyema of each patient were recorded. Postoperative outcomes, including morbidity and mortality, length of hospital stay (LOS), and pre- and post-treatment empyema cavity volume, as measured by CT-volumetry, were investigated. The LOS and mortality were compared with data from our historical cohort of patients treated with VATS or open thoracotomy.

The primary endpoint of the study was therapeutic success rate, as defined by infection resolution and no need for further empyema-related medical or surgical treatment following FlexVATS treatment.

### Operative technique

#### I. First procedure

Following the diagnosis of pleural empyema, either sonographic or CT-guided drainage placement was performed as first-line treatment in combination with empiric antibiotic therapy. Surgery was performed 1–4 days following drainage placement, depending on the condition of the patient, with septicaemia warranting earlier surgical intervention, while stable patients were surgically treated after treatment initiation via drainage and antibiotic therapy.

At the first surgical intervention, all patients received a double-lumen tube or bronchial blocker, so the relevant lung could collapse by suction if needed. The procedure was performed with the patient in the lateral decubitus position.

**Figure 1:**
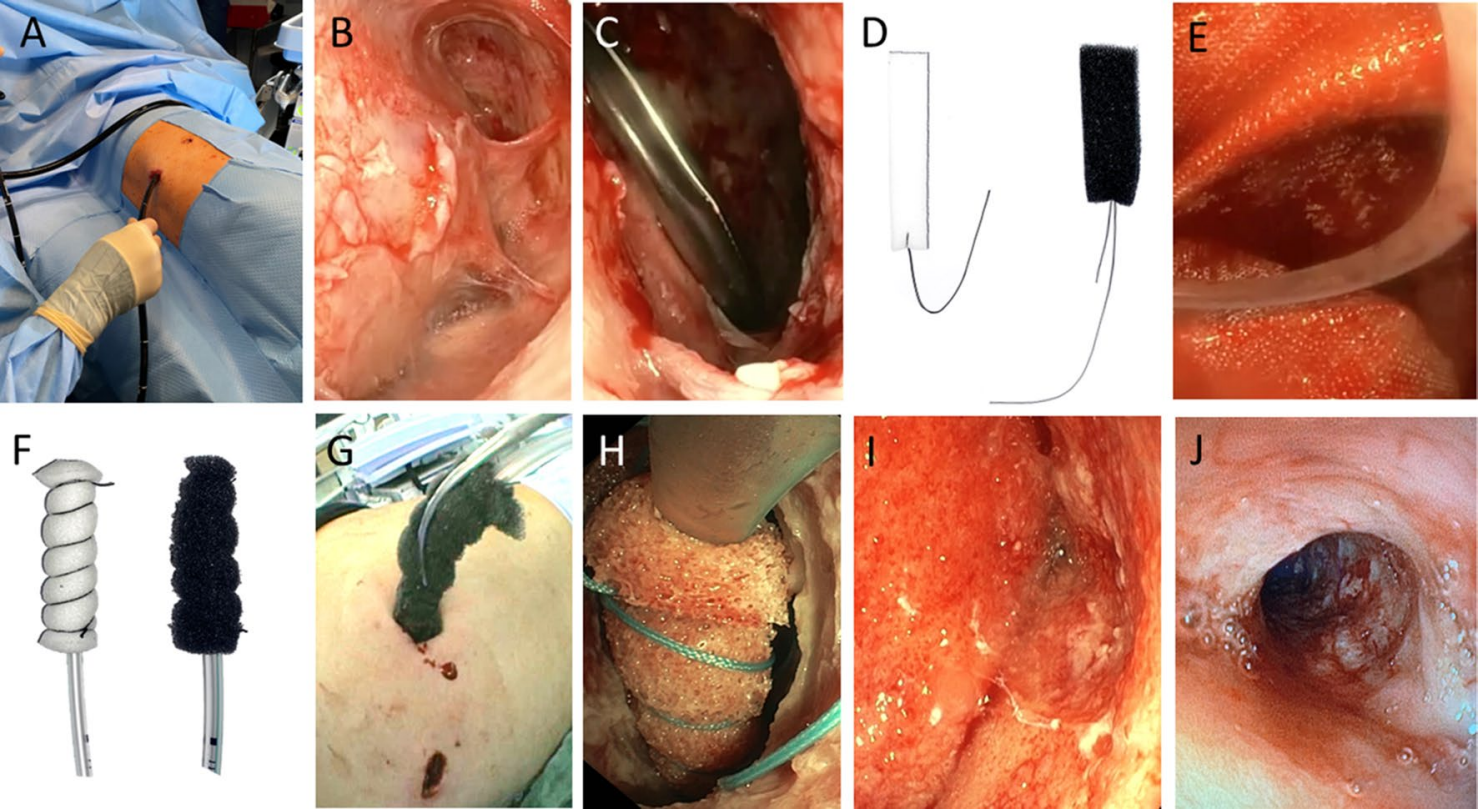
Step-by step approach for FlexVATS technique. (**A**) Operative Setting using a flexible endoscope. (**B**) Intraoperative view of fibrin und inflammatory tissue in the pleural space, (**C**) blunt debridement and removal with a large forceps. (**D**) Examples of white and black sponge segments with non-absorbable sutures. (**E**) Open pore film and rinsing drainage insider the pleural cavity. (**F**) Examples of white and black ‘endo-sponges’, introduction via the 2–3 cm incision. (**H**) intraoperative view of a white ‘endo-sponge’. (**I**) Good granulation tissue in the complete empyema cavity after several dressing changes. (**J**) Final view after removal of an open pore film drainage.

First, the drainage channel was widened to safely reach the empyema cavity, and an endoscope [usually a standard gastroscope (8–10 mm wide)] was introduced (Fig. 1A). The cavity was inspected, and an endoscope was used to bluntly open the empyema septations (Fig. 1B). Under direct vision, one or two incisions were made, and 10 mm trocars were inserted. Using either laparoscopic or curved instruments, including a wide suction cannula, the septic and fibrinous material was loosened, bluntly abraded, and removed (Fig. 1C). Importantly, no pleurectomy was performed and injury to the visceral pleura was strictly avoided. Thorough rinsing with saline and precise haemostasis were then performed.

The second step was the insertion of black or white sponge segments (3M™ V.A.C. Granufoam™, 3M St. Paul, MIN, or V.A.C. WHITEFOAM™ Small Dressing) or open pore film material (Suprasorb CNP by Lohmann & Rauscher, Germany). The sponges were cut into long cuboids that could be inserted through mini-thoracotomy, with extracorporeal fixation of the ends using a non-absorbable suture for easier extraction (Fig. 1D)^17,18^. Black sponge material was mainly used in the first step because of its superior effectiveness in tissue cleaning and granulation compared with other existing sponge materials. White foam material was indicated only in cases of relatively clean cavities or if the interval to the next changing procedure needed to be longer than 3–5 days due to the patient’s condition or organisational reasons. It proved vital that the thoracic wall and empyema wall had contact with sponge material. In the case of large cavities with a size over 15 × 15 cm, an open pore film was inserted, unfolded and the pleural cavity was covered (Fig. 1E). For intrathoracic application of negative pressure, an endovac was manufactured and inserted (Fig. 1F–H). The negative pressure was applied via an electric pump (3M, St. Paul, MIN) with a value of −125 mmHg.

#### II. Following interventions/ dressing changes

When a black sponge material was used, dressing changes were performed after 3–5 days. In the case of the white sponge material and open-pore film, the material was changed after 3–8 days. For dressing changes, less extreme positioning is required (usually in the supine or half-lateral position). Patients were either intubated or treated in analgo-sedation, depending on the comorbidities of the patient and risk assessment of the anaesthesiologists. The suction tube (endosponge) was removed, and an endoscope was inserted. The dressing material was removed using direct vision. The progress was evaluated and the cavity was rinsed with saline. Depending on the grade of granulation of the empyema membrane and the macroscopic cleanliness (purulent and/or fibrinous debris) of the cavity, the next procedure was planned according to the suction material chosen. In cases of slow progress, black sponges were repeatedly used. If good granulation was observed, the size of the inserted material was reduced, white sponge material was inserted in the regions where needed, and the therapy continued (Fig. 1I). With each intervention, the size of the material was reduced to induce an adhesion of the granulated visceral and parietal pleura. In case of a clean wound with a proper granulation and a recovered lung flexibility to re-expand by suction and complete collapse of the cavity, the therapy was discontinued (Fig. 1J). A normal drainage or a subcutaneously or intercostal suction sponge was inserted during the last intervention and removed after 5–7 days without sedation in an outpatient setting in cases where skin closure was not possible during the final dressing removal.

Treatment success was monitored clinically using routine laboratory parameter analysis. In cases of systemic signs of infection, a CT scan was performed to identify parts of the empyema that were not adequately treated.

### CT volumetric analysis

**Figure 2:**
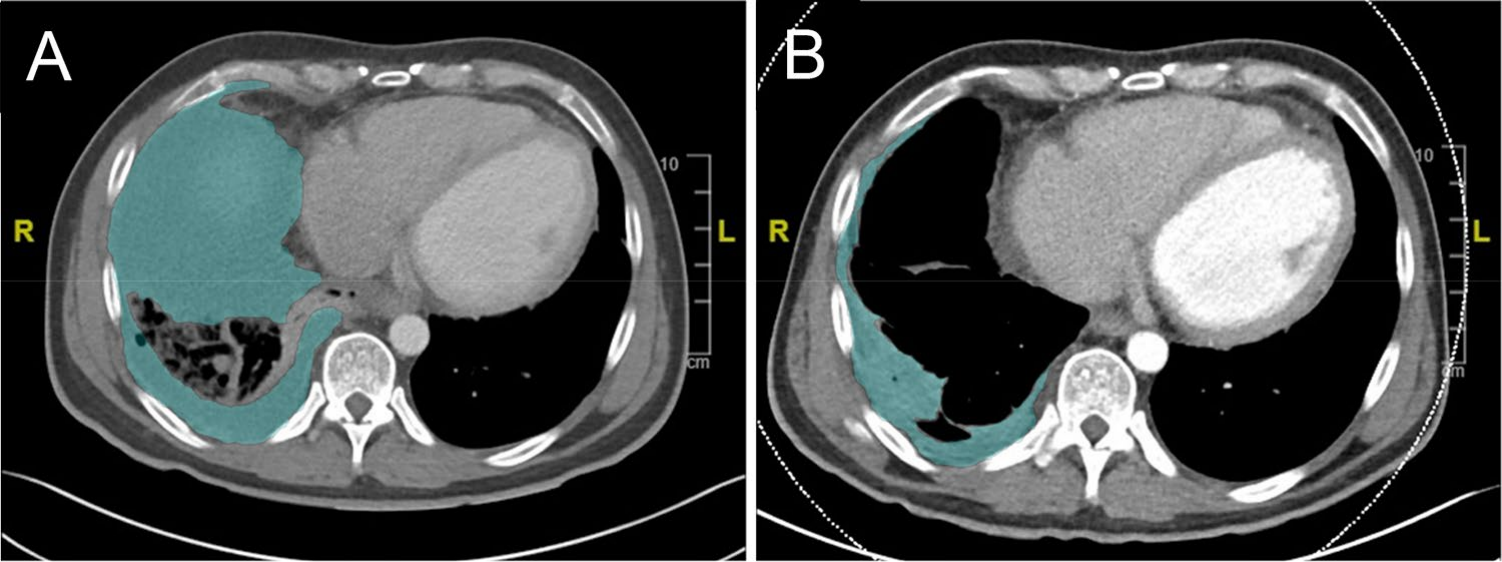
Pre- and post-treatment CT-volumetry of the empyema cavity. Exemplary transverse plane CT imaging with the borders of the empyema cavity shaded in green: pre- (**A**) and post-treatment (**B**).

Computed tomography scans were performed on a clinical 64-detector CT scanner, mostly in the portal venous phase (80% portal venous, 12% native, and 8% arterial). The data were reconstructed in the transverse plane with a slice thickness of 1 mm and an increment of 1 mm. Volumetry was performed by an experienced radiologist (J. S.) using a dedicated DICOM Viewer (Centricity Universal Viewer Version 6.0, GE Healthcare, Munich). Volumetric measurements of pleural empyema were calculated using the DICOM viewer in a semiautomatic fashion, where borders were marked manually in the transverse plane on each slice (Fig. 2). Subsequently, the software automatically calculated the volume^20^.

### Statistical analysis

Statistical analysis was performed using IBM SPSS ver. 24 (Armonk, NY, USA). The Shapiro–Wilk test was used to test for the normality of distribution. Continuous variables with normal distribution are reported as mean ± standard deviation (SD), and non-normal variables are reported as median + interquartile range. To compare the preoperative and postoperative volume of the empyema cavity, the Wilcoxon matched pair signed rank test was used. A comparison between the volume reduction in this study and that of Nishi et. al^21^ was performed using the Mann–Whitney-U test. The LOS and 90-day mortality rates for FlexVATS, VATS, and thoracotomy were compared using the Kruskal–Wallis test, followed by Dunn’s test for pairwise multiple comparisons of the ranked data.

A *p*-value ≤ of 0.05 was considered significant.

### Ethics statement

All procedures performed in studies involving human participants were approved by the Ethics Committee of the University of Hamburg, Germany in accordance with the ethical standards of the institutional research committee and with the 1964 Helsinki Declaration and its later amendments or comparable ethical standards (protocol code: PV3548).

### Informed consent

All patients gave written informed consent before being included in the study.

## Results

### Baseline demographics and comorbidities

Between February 2021 and August 2022, 17 patients were treated using FlexVATS. The median (IQR) age was 66 years (46–79) years. There was a male predominance across the cohort (4.6:1) (Table 1). More than half (53%) of the patients had ≥ 2 comorbidities, indicating that this study included many multimorbid patients (Table 2). Only four (24%) patients had no comorbidities. Three of the 17 patients (18%) were admitted to the intensive care unit (ICU) prior to FlexVATS treatment.

**Table 1:** Baseline demographics, number of comorbidities and cause of empyema.

No	Sex	Age, years	No. of comorbidities	Cause of empyema
1	M	87	1	Pneumonia
2	M	41	4	Pneumonia
3	M	61	1	Pneumonia
4	M	44	3	Infectious pleural effusion
5	M	48	0	Pneumonia
6	M	80	4	Infectious pleural effusion
7	M	82	4	Infectious pleural effusion
8	M	78	3	Infectious pleural effusion
9	M	66	1	Chronic empyema
10	M	20	2	Pneumonia
11	F	68	0	Pneumonia
12	F	49	1	Infectious haemothorax
13	M	68	3	Infectious pleural effusion
14	M	86	2	Infectious pleural effusion
15	M	63	4	Pneumonia
16	M	20	0	Infectious haemothorax
17	F	69	0	Pneumonia

*Y* years, *F* female, *M* male.

**Table 2:** Comorbidities and their prevalence within the study cohort.

Comorbidity	Prevalence (%)
Chronic heart failure	10 (59)
Chronic liver disease	1 (6)
COPD	2 (12)
Diabetes	2 (12)
Alcohol abuse	1 (6)
Malignancy	1 (6)
Smoking	4 (24)
Renal insufficiency	6 (35)
Immunosuppression	1 (6)

*COPD* chronic obstructive pulmonary disease.

### Cause of empyema

Primary empyema was present in five (29%) patients, with eight (47%) patients having parapneumonic empyema (Table 1). Four patients (24%) developed empyema secondary to thoracic surgery. Thoracic surgery included one case of recurrent empyema after pneumonectomy, one case of open resection of a large bronchogenic cyst, one case of VATS hematoma evacuation of a haemothorax, and one case of empyema recurrence following VATS decortication.

### Treatment-related data

**Figure 3:**
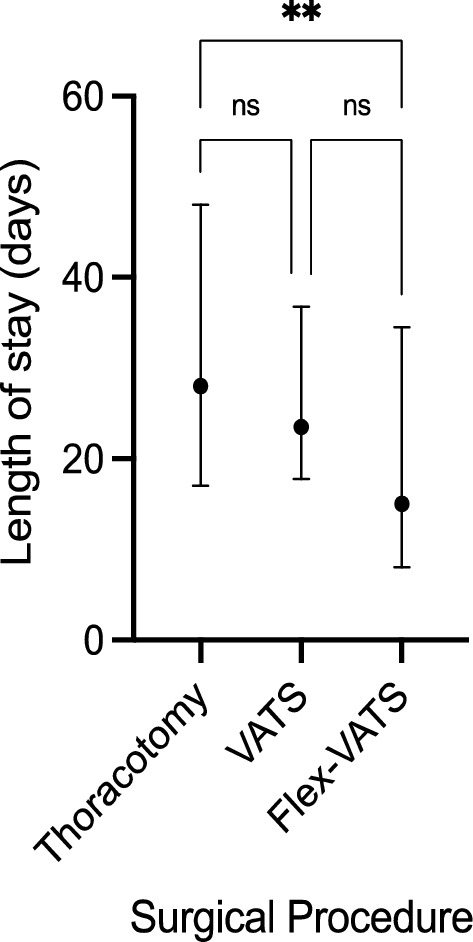
Length of stay for FlexVATS cohort compared to thoracotomy and VATS. Data are presented as median ± interquartile range. Statistical significance was assessed using the Kruskal–Wallis test followed by Dunn’s test for pairwise multiple comparisons of the ranked data. Statistical significance was set at P < 0.05. **Significant difference (p = 0.002); *ns* not significant. *VATS* video-assisted thoracoscopic surgery, *FlexVATS* flexible video-assisted thoracoscopic surgery.

The median (IQR) LOS for patients who underwent FlexVATS was 15 days (8–35 days). We compared these data with those of patients treated between 2017 and 2022 at our institution with thoracotomy or VATS (n = 181 and 42, respectively). For patients treated with thoracotomy, the median (IQR) LOS was 28 days (17–48 days). The median (IQR) LOS for patients treated with VATS was 24 days (18–37). When comparing the FlexVATS to the complete cohort, a trend in reduction of LOS was observed when compared to the VATS group; however, this difference was not significant (p = 0.05, z-score 2.4). Compared with thoracotomy, the difference in LOS for patients in the FlexVATS group was significantly reduced (p = 0.002, z-score 3.5). The results are shown in Fig. 3.

In total, a median (IQR) of 3 (2–6) dressing changes was required (Table 3). The median (IQR) negative pressure was 125 (113–125) mmHg. 27 out of 66 (41%) vacuum dressing changes were possible outside of the operating room without the need for general anaesthesia.

**Table 3:** Treatment data and patient’s outcome.

No	Duration, days	Vacuum, mmHg	No. of dressing changes		Outcome
			Inside OR (%)	Outside OR (%)	
1	18	100–150	2 (50)	2 (50)	Successful treatment
2	36	125	3 (50)	3 (50)	Successful treatment
3	15	125	1 (33)	2 (67)	Successful treatment
4	43	125	5 (39)	8 (62)	Death (cardiac failure)
5	36	125	1 (14)	6 (86)	Successful treatment
6	35	75–125	4 (67)	2 (33)	Successful treatment
7	1	125	0 (0)	0 (0)	Death (septic shock)
8	8	125	2 (100)	0 (0)	Successful treatment
9	34	125	8 (100)	0 (0)	Open window thoracotomy
10	1	100	0 (0)	0 (0)	Death (septic shock)
11	2	100	1 (100)	0 (0)	Successful treatment
12	15	150	1 (25)	3 (75)	Successful treatment
13	8	125	1 (50)	1 (50)	Successful treatment
14	8	125	2 (100)	0 (0)	Open window thoracotomy
15	16	125	4 (100)	0 (0)	Successful treatment
16	15	125	2 (100)	0 (0)	Successful treatment
17	9	125	1 (100)	0 (0)	Successful treatment

*OR* operating room.

### Outcomes

Six of the 17 patients (35%) were postoperatively treated in the ICU, with a median (IQR) ICU stay of 9 (11–303) days. The mean (SD) follow-up time was 76 days (119).

Infection resolution without the need for ongoing antibiotic or drainage therapy was achieved in 12 of the 17 (71%) patients. Nine of the 17 patients (53%) were discharged with secondary wound healing of the mini-thoracotomy wound. Three patients required chest tube treatment for a short period following the removal of the vacuum dressing, and all drains were removed before discharge. No vacuum-related complications, such as bleeding or air leakage, were observed. One patient with an otherwise successful course of treatment developed severe periprocedural stroke, which was deemed a non-treatment-related event.

Three of the 17 patients (18%) showed multi-organ failure due to septic shock prior to FlexVATS treatment, with all three patients dying during the hospital stay. These patients were all multimorbid and had severe pre-existing conditions (diabetes mellitus, chronic heart and kidney failure, and immunosuppression), making them poor candidates for surgery. Table 3 summarizes the treatment outcomes.

**Figure 4:**
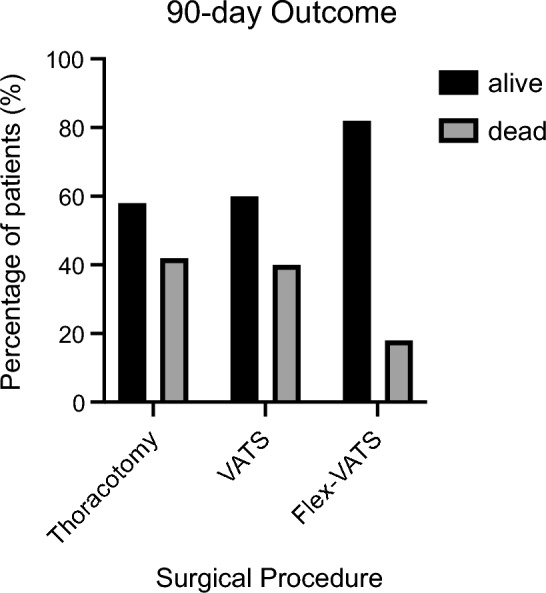
90-day mortality rates for FlexVATS cohort compared to thoracotomy and VATS. There was a trend in reduction of 90-day mortality rates without reaching statistical significance. Statistical significance was assessed using the Kruskal–Wallis test followed by Dunn’s test for pairwise multiple comparisons of the ranked data. Statistical significance was set at P < 0.05.

Patients treated with FlexVATS showed a trend towards reduced 90-day mortality rates compared with VATS or open surgical decortication in the historical cohort (18% versus 41% and 42%, respectively, p = 0.15, Fig. 4).

### CT-volumetry

Sixteen patients underwent at least one CT scan, 65% (11/17) before and after intervention, and 30% (5/17) only before the intervention. Patients had a median (IQR) empyema cavity volume of 310 (191–798) cm^3^ before vacuum therapy and 37 (25–323) cm^3^ after vacuum therapy (Table 4). Empyema cavity volume significantly decreased with FlexVATS therapy (p = 0.008). In comparison to the VAC following the OWT cohort described by Nishii et al., there was no significant difference in relative volume reduction of the empyema between our results and those of Nishii et al. (p = 0.09)^21^.

**Table 4:** Results of CT analysis of empyema cavity volume.

No	Preoperative volume, cm^3^	Postoperative volume, cm^3^	Relative volume difference, cm^3^ (%)
1	496	13	− 483 (97)
2	850	32	− 818 (96)
4	620	100	− 520 (84)
5	114	25	− 89 (78)
6	213	15	− 198 (93)
9	950	323	− 627 (66)
12	334	123	− 211 (63)
13	1079	873	− 206 (19)
14	183	389	+ 206 (113)
15	280	37	− 243 (87)
17	120	27	− 93 (78)

## Discussion

We present a series of 17 patients who underwent minimally invasive intrathoracic vacuum treatment with flexible endoscopy. The majority (70%) of the cases were successfully treated, with moderate treatment-related morbidity and mortality.

The more sparing therapeutic approach, treating only the affected part of the lung and moving away from extensive decortication, showed an acceptable postoperative morbidity and in our opinion explains why in this study, no significant air leaks or haemorrhage (intra- or postoperatively) occurred in the FlexVATS group. In comparison, in VATS versus open surgical decortication, the leak rate is in the published literature approximately 17 vs. 16% and haemorrhage occurs in 6 vs. 5% of cases^22^.

A comparison of the treatment outcomes of FlexVATS with the currently available surgical approaches is promising. We demonstrated a significantly reduced LOS in our cohort compared to patients treated with thoracotomy at our institution (p = 0.002). When comparing FlexVATS to conventional VATS, a trend in reduction of LOS was observed; however, this difference was not significant (p = 0.05). Compared with thoracotomy and VATS, patients treated with FlexVATS showed a reduction in 90-day mortality rates without reaching statistical significance (42% vs. 41% vs. 18%, p = 0.15).

It does appear necessary to comment on the relatively high mortality rates in the thoracotomy and VATS groups which we used as a comparison to our FlexVATS group. The advantage of our treatment strategy was that it could be performed without single lung ventilation and was therefore especially advantageous for very ill patients. These patients had been historically treated at our institution with thoracotomy or VATS as a rescue procedure if conservative treatment with pleural drainage had failed. Additionally, a fairly large subgroup in the historical cohort had empyema due to lung surgery or oesophageal surgery, and most existing studies investigate mortality rates in patient cohorts with primary empyema^23,24^.

Apart from infection resolution defined by missing signs of systemic infection and the need for ongoing antibiotic therapy or drain placement, we added CT-volumetry before and after treatment as an objective parameter for evaluating treatment efficacy. Nishii et al. reported the use of lung CT volumetry as a treatment success parameter for vacuum therapy in patients with OWT and demonstrated an approximately 60% volume reduction in these patients. In comparison to the cohort described by Nishii et al.^21^, there was no significant difference in the relative volume reduction of empyema treated with OWT and our results for the primary treatment of empyema with FlexVATS (p = 0.09). Furthermore, we demonstrated a significant reduction in empyema cavity volume by applying the FlexVATS method as a primary treatment method for pleural empyema. Our patients showed a median empyema cavity volume of 310 cm^3^ before versus 37 cm^3^ after vacuum therapy, with a significant reduction (p = 0.008).

As already mentioned, intrathoracic vacuum therapy has been described mostly in patients with OWT, with only a few small series, but the data have shown much promise as a first-line treatment option for empyema^6,15,16,25,26^.

Vacuum therapy has been established for a variety of wounds, and different dressing materials can easily be adapted for intrathoracic use. Several materials, such as open pore films and small pore sponges, have been developed to tailor optimal therapy for a variety of purposes. Open pore film drainage has been used either extraluminal for small gastrointestinal leaks or intraluminal and can be easily placed in the thoracic cavity to ensure adequate cleaning and granulation of the empyema cavity^19,27^.

Endoscopic techniques using flexible endoscopes for vacuum-material applications have already been developed and presented in previous publications^18^. We refined this technique to allow minimally invasive thoracic application without the need for single-lung ventilation, carried out in analgo-sedation for dressing changes after the initial VAC application.

In this patient series presenting a new treatment method for patients with localized pleural empyema, FlexVATS was offered as a treatment alternative to both healthy and multimorbid patients. However, it needs to be investigated whether the therapy might have a role in late stages with its high conversion rate to open surgery or even in earlier empyema. To date, this technique has been used in patients with localized, defined, and capsuled empyema with basal and/or dorsal extension. Potential contraindications may be patients with an therapeutic anticoagulation due to clotting of the sponge or tubes due to haemorrhaging after the procedure, and iatrogenic or pre-existing pulmonary air leaks that lead to misfunctioning of the negative-pressure pump. In early stages of empyema or cases with a complete pleural manifestation, the method might not be applicable and conventional minimally invasive procedures should be used.

A known drawback of negative-pressure therapy is the need for repeated procedures with sedation and its possible complications and stress for patients. While the first operation was carried out in lateral decubitus positioning and general anaesthesia, as well as a double-lumen tube or bronchial blocker to allow for the collapse of the treatment side if needed, the subsequent interventions were less time-intensive and could be carried out in—depending on the morbidity of the patients—in analgo-sedation with less extreme patient positioning. This proved to be an excellent treatment option that could be offered even to the most debilitated patients who would otherwise not tolerate repeated operations. However, in the last 20 years using endoscopic vacuum therapy, the method is safe in experienced hands and does not cause elevated harm to patients. We are optimistic that with increasing experience, the first operation can also be safely performed without double-lumen tubes or bronchial blockers.

This study has several limitations. The single-institution design and relatively small cohort limit the conclusions that are applicable to all patients with pleural empyema. After demonstrating the efficacy of this advanced treatment method, we hope that a larger study across multiple institutions will validate this method.

In conclusion, this case series demonstrates a modified treatment technique for patients with pleural empyema. FlexVATS therapy shows promising success rates with significantly reduced LOS compared with open surgery and high rates of infection resolution. There was a significant reduction in cavity empyema volume, as measured by computed tomography volumetry. The 90-day mortality rates were significantly lower than those of VATS and thoracotomy. The procedure can be carried out in analgo-sedation and without single lung ventilation. This makes it an excellent treatment option for patients who are poor surgical candidates who were previously not amenable to surgical therapy. Larger-scale comparative trials are needed to confirm that this treatment option is another pillar in the treatment of pleural empyema.

## Data Availability

Research data supporting this publication are available upon request from the corresponding author.
